# Burden and determinants of multimorbidity among women in reproductive age group: a cross-sectional study based in India

**DOI:** 10.12688/wellcomeopenres.16398.1

**Published:** 2020-11-24

**Authors:** Parul Puri, Ajinkya Kothavale, S.K. Singh, Sanghamitra Pati

**Affiliations:** 1 International Institute for Population Sciences, Govandi Station Road, Deonar, Mumbai, Maharashtra, 400088, India; 2 ICMR Regional Medical Research Centre, Indian Council of Medical Research, Department of Health Research, Bhubaneswar, Odisha, 751023, India

**Keywords:** multiple chronic condition, multimorbidity, non-communicable diseases, obesity, body-mass index, women, India

## Abstract

**Background:** India's government is currently running several programs with a sole focus on women's health during their child-bearing years. However, none of these programs incorporate the management of chronic health conditions during the reproductive span. This issue is an emerging public health concern; therefore, the present study aims to identify the patterns and correlates of multimorbidity among women in reproductive age groups in India.

**Methods:** The study utilizes nationally-representative cross-sectional data from the Demographic and Health Survey on 661,811 women in the reproductive age group of 15-49 years. The study uses information on seven chronic morbidities, namely asthma, cancers, heart disease, diabetes, tuberculosis, hypertension, and thyroid disorder. Descriptive, bivariate, and multivariable techniques were utilized to accomplish the study objective.

**Results:** The findings show that 17.4 and 3.5 per 100 women of reproductive age suffered from any one morbidity and multimorbidity, respectively. Hypertension, diabetes, and thyroid disorders were commonly occurring morbidities. The prevalence of having any one morbidity or multimorbidity increased with age. Variables like religion, wealth, parity, menopause, consumption of tobacco and alcohol, body mass index, and type of diet were found to be significantly related to the burden of multimorbidity. The prevalence of multimorbidity was found to be higher for women who belong to the Southern, Eastern, and North-Eastern regions of India.

**Conclusions:** Findings suggest the importance of multimorbidity in the context of women of reproductive age. Inclusion of chronic disease management strategies with maternal and child health services needs to be taken into consideration by the program and policymakers. The annexation of social marketing approaches at the primary level of healthcare would assist policy-makers in educating women about the importance of leading a healthy lifestyle. Practicing dietary diversity can help in maintaining optimal estrogen levels, which would further help in decreasing multimorbidity rates among women in India.

## Introduction

As per the World Bank estimates, the life expectancy in India has increased from 42.27 years in 1960 to 69.16 years in 2017 ( World Health Organization, 2020a), with women currently outliving men. However, these increased years of life do not necessarily ensure better health. The World Health Organization (WHO) establishes that the sex of an individual plays a significant role in determining their health-related outcomes ( World Health Organization, 2020b). The reason for this difference is both biological and gender-related ( World Health Organization, 2020b). Globally, women face discrimination because of deep-rooted gender inequality ( Davidson *et al.*, 2011). This inequality results in an uneven distribution of work, with women taking up primary caregivers’ responsibility in most societies ( AMCHP, 2008). The issue becomes severe in developing countries, like India, where the women fall into the category of vulnerable members of society, with only a low percentage participating in economic activities and decision making ( Verick, 2011; Yadav & Singh, 2020). This disparity results from of a wide variety of social, economic, and developmental influences in the country ( Verick, 2011; Yadav & Singh, 2020). Women in India are, therefore, at higher risk of living with poor health outcomes. These poor health outcomes can be seen as a result of the different events in a woman’s life course ( Lynch, 2003). One of the critical events in a woman’s life course is the occurrence of menarche, which announces a women’s entry into her reproductive cycle. WHO defines the age group of 15–49 years as the reproductive span in a woman’s life ( World Health Organization, 2020c).

To reduce gender inequality and build a more sustainable future for all, the third objective of the Sustainable Development Goals (SDGs) ensures healthy lives and promotes well-being for all ages ( United Nations, 2020). The targets set by the SDG regarding women’s health focus mainly on improving outcomes related to sexual and reproductive healthcare among women. Women in India spent around half of their life span in their reproductive years ( World Health Organization, 2020a). Thus, reproductive health has been a priority since the initiation of the Reproductive Child Health Program in India during 1997 ( National Health Portal, 2015). There are several programs, like Pradhan Mantri Matritva Vandana Yojana ( Government of India, 2017), Pradhan Mantri Surakshit Matritva Abhiyan ( Government of India, 2016), and Janani Suraksha Yojana ( National Health Portal, 2005) presently running with a sole focus on women’s health during their child-bearing years in India. However, these programs do not include provision of screening and management of chronic morbidities among women in the reproductive age group. 

Studies done in different developed countries propose that women of reproductive age are at higher risk of being affected by one or more chronic diseases ( AMCHP, 2008; Waller *et al.,* 2018). Morbidity burden has severe health implications on women and child health in both the early and later stages of life ( AMCHP, 2008). In addition, the reproductive period and conception are the first points of interaction of women with the health care infrastructure in India ( AMCHP, 2008). Thus, ensuring proper disease management during the reproductive years becomes obligatory.

 Existing literature describes the linkage between socioeconomic status (SES) and behavioral influences on the occurrence of one or more chronic morbidities in several country settings over all ages ( Banjare & Pradhan, 2014; Tetzlaff *et al.*, 2018). Further, these studies establish the significance of exploring multimorbidity as an independent domain, due to its accelerating burden and association with unfavorable health outcomes, like declining functional status, low levels of social interaction, poor quality of life, low satisfaction level, higher mortality risks, increased healthcare utilization and increased economic burden on the patients’ household ( Braveman *et al.,* 2005; Boyd & Fortin, 2010; Marengoni *et al.*, 2011).

 Despite an enormous body of literature that discusses the importance of multimorbidity, there are fewer studies that focus on linking the influence of reproductive exposures on the occurrence of chronic morbidities and multimorbidity. Additionally, none of India’s programs currently incorporate management of chronic health conditions during the reproductive span (15–49 years). Thus, it becomes essential to explore the domain holistically, keeping reproductive-age women central, to inform community-oriented health-related programs and policies ( Xu *et al.,* 2020). Therefore, this study aims to identify the patterns of one or more morbidities and to examine the correlates of multimorbidity among women in the reproductive age group (15–49 years) in India.

## Methods

### Ethical statement

The study utilizes data from a national survey conducted under the stewardship of Ministry of Health & Family Welfare, Government of India, with the help of International Institute for Population Sciences, Mumbai. The survey received ethical clearance from the Institutions Review Board (IRB) of International Institute for Population Sciences, Mumbai, India. Additionally, consent was taken from all the eligible participants above the age of 18 years. For children (6–59 months), consent was obtained from a parent or guardian. NFHS data has been archived in the Demographic and Health Surveys’ public repository. As the data is freely available for research purposes, no additional permissions were required to conduct the present analysis.

### Data

The present study utilizes the data from the fourth round of the National Family Health Survey (NFHS), India, 2015–16. NFHS is a cross-sectional population-based survey whose primary objective is to provide national and sub-national level estimates of the data on population, health, nutrition, and other key demographic indicators for India. The evidence generated by NFHS abets the policymakers in establishing benchmarks, evaluating the effectiveness of currently running programs, and identifying the need for new programs in the areas specific to family and health. The sampling design adopted by NFHS-4 was a two-stage stratified sampling considering urban and rural areas as the natural strata ( International Institute for Population sciences, 2015).

 NFHS-4 collected data from a nationally representative sample of 699,686 women in the age group 15–49 years, out of which the study utilized information on 661,811 women (15–49 years) who were not currently pregnant. The primary reason for including non-pregnant women is to ensure a common cut-off for body mass index (BMI). The information on women was collected from all 36 states/Union Territories (UTs) of India ( International Institute for Population sciences, 2015).

### Variables

The study aims to identify the patterns and factors affecting the burden of multimorbidity among women in the reproductive age group of 15–49 years in India. Existing literature proposes multidimensional linkages between SES ( Braveman *et al.*, 2005; Banjare & Pradhan, 2014), reproductive exposures ( Gold, 2011; Schmidt, 2017; Yang *et al.*, 2017) and behavioural factors ( Goel *et al.,* 2014; Mini & Thankappan, 2017) with one or more morbidities. It is therefore crucial to incorporate all available and feasible individual-level indicators of SES, reproductive exposures and behavioral risk factors in this study.

***Socioeconomic and demographic variables.*** The variables included under this heading are age (15–19 years; 20–24 years; 25–29 years; 30–34 years; 35–39 years; 40–44 and, 45–49 years), place of residence (rural; urban), religion (Hindu; Muslim; Others), social group (Scheduled Castes/Tribes; Other Backward Classes (OBC) and Others), level of education (no education; primary; secondary and, higher) and, wealth index (poorest; poorer; middle; richer, richest).

It is worth mentioning that variables like social group and religion are included in the study as they are building blocks of Indian society and thus play a significant role in defining the SES of a respondent ( Goli *et al.,* 2016). Information on income or expenditure is not collected in the NFHS. Therefore, the wealth index is utilized to measure the SES of the respondent. Existing literature suggests the advantages of using the DHS wealth index (computed using the information available on assets and amenities) to measure the SES holistically ( Filmer & Pritchett, 2011; Rutstein & Johnson, 2004).

***Reproductive exposures.*** Three variables were included under this heading, namely family status (living without spouse; living with spouse), parity (nulliparous; one, two or more), and experienced menopause (no; yes) ( Xu *et al.*, 2020; Yang *et al.*, 2017). In order to avoid issues of multicollinearity, other variables related to reproductive exposures are not included in the present study.

***Lifestyle variables.*** This included behavioral risk factors like consumption of tobacco (no; yes) ( Goel *et al.*, 2014; Mishra *et al.*, 2016), consumption of alcohol (no; yes) ( Murthy *et al.,* 2010; Neufeld *et al.,* 2005), BMI (underweight; normal; overweight; obese) ( NCD Risk Factor Collaboration (NCD-RisC), 2017; Jovic *et al.,* 2016; Ramdas *et al.,* 2018) and, type of diet (healthy; unhealthy) ( Joy *et al.*, 2017; Kumar *et al.,* 2014; Shridhar *et al.,* 2015).

NFHS collects information on the frequency (daily, weekly, occasionally, and never) of consuming nine food items, namely milk/curd, pulses/beans, dark leafy vegetables, fruits, eggs, fish, chicken/meat, fried food, and, aerated drinks. All these nine food items were re-coded in order to make them one-dimensional, such that each item measures the same concept. The internal consistency was supported using Cronbach’s alpha ‘α’. Type of diet was computed utilizing a multiple correspondence analysis (MCA) as suggested by existing studies done in India ( Mishra & Monica, 2019). Further, the score generated was categorized to form type of diet variable: healthy or unhealthy.

***Outcome.*** For analysis, two outcome variables have been utilized to measure the level of multimorbidity, namely, the presence of two or more chronic health conditions (multimorbidity) and the number of chronic health conditions present in an individual.

To calculate the number of chronic conditions present in an individual, information available from both self-reported and clinically diagnosed data is used. The study incorporates all the seven chronic conditions, namely, asthma, cancers, heart disease, diabetes mellitus, tuberculosis, hypertension, and thyroid disorder, available in the NFHS-4 data ( International Institute for Population sciences, 2015). Detailed information on the chronic conditions included in the study are provided in Table 1.

**Table 1.** List of the chronic conditions included in the study with their mode of data collection and ICD-10 codes.

Morbidities	ICD 10 codes	Data collection mode
Asthma	J40-J45	Self-reported
Cancers	C00-C14, C15-C26, C30-C39, C40-C41, C43-C44, C45-C49, C50, C51-C58, C60-C63, C64-C68, C69-C72, C73-C75, C81-C96, C76-C80, C97, D00-D09, D37-D48	Self-reported
Heart disease	I20, I21, I25	Self-reported
Diabetes	E10-E14	Self-reported and measured diagnoses
Hypertension	I10-I15	Self-reported and measured diagnoses
Thyroid	E01-E05, E06.1-E06.3, E06.5, E06.9, E07	Self-reported
Tuberculosis	A15, A17, A18, A19	Self-reported

 Data on diabetes mellitus and hypertension were collected utilizing information from both self-reported data and clinically diagnosed results. NFHS-4 classified hypertension (blood pressure), by taking into account various combinations of systolic and diastolic measurements. To attain greater accuracy, the readings for both systolic and diastolic blood pressure were taken thrice with an interval of five minutes between two consecutive readings using an Omron Blood Pressure Monitor. For the present analysis, a respondent was considered to be hypertensive if the average systolic blood pressure was greater than or equal to 140 mmHg or average diastolic blood pressure was greater than or equal to 90 mmHg or the individual disclosed current intake of any anti-hypertensive medicines (NFHS-4) ( International Institute for Population sciences, 2015).

The measurement of diabetes was done utilizing random blood glucose level, which was measured using a FreeStyle Optium H glucometer with glucose strips. The equipment uses a finger stick blood specimen to provide the random blood glucose reading. An individual was considered as diabetic if their random blood glucose level was greater than or equal to 140 mg/dL or they reported taking medications for lowering blood glucose level (NFHS-4) ( International Institute for Population sciences, 2015).

BMI was calculated utilizing the anthropometric measurements height and weight. Height was measured using a Seca 213 stadiometer and weight was measured using Seca 874 digital scale. The formula for computing BMI is as follows:

$$BMI=\frac{Weight\;(in\;kgs)}{Height\;(in\;metres^{2})}$$

An individual was considered to be underweight if their BMI was less than 18.5 kg/m ^2 ^, normal if their BMI was between 18.5–24.9 kg/m ^2^, overweight if their BMI was between 25.0 to 29.9 kg/m ^2, ^and obese if their BMI was greater than or equal to 30 kg/m ^2^ ( International Institute for Population sciences, 2015). All the cut-offs selected are in sync with the levels proposed by the Demographic Health Surveys (NFHS-4) ( International Institute for Population sciences, 2015).

### Statistical analysis

Descriptive statistics followed by bivariate analyses were used to examine the unadjusted association between the selected exposure variables and the outcome of interest, which in this case is the presence of multimorbidity. Prevalence of any one morbidity and multimorbidity was computed by all the selected background characteristics.

The results from the primary analysis depict that a significant share of the surveyed population did not suffer from multimorbidity. Therefore, the distribution of the outcome of interest is positively skewed. In order to solve this issue, a two-stage estimation procedure, like the two-part model, is frequently used. The two-part model is often used to model strictly positive variables with a large number of zero values. This model was consequently formulated as a mixture of a binomial distribution and a strictly positive distribution. The two-part model is commonly used in health economics studies to model healthcare expenditure data because a large fraction of patients do not spend anything on medical care in a given time ( Buntin & Zaslavsky, 2004; Kapitula & Valley, 2015).

The first stage defines the outcome as a dichotomous variable, which, in this case, is multimorbidity (present=1, absent=0). This part can be referred to as the ‘prevalence part’. After completion of the first stage, which group of dependent variables the observations belong to is identified. The second stage considers the number of morbidities (count data) if the selected respondent has the outcome of interest, i.e., multimorbidity. Therefore, to predict the above situation a two-part model is utilized, considering it as a mixture of two distributions, one consisting of a point mass at zero values, followed by a truncated count data distribution for the non-zero observations. Thus, for addressing the issue in hand, for the first part, logistic link function would be applied (considering multimorbidity as a dichotomous variable; present=1, absent=0), followed by a generalized linear model using Poisson regression ( Buntin & Zaslavsky, 2004; Kapitula & Valley, 2015).

The present analysis was done using Stata version 15.0 (Stata Corp Inc. TX, USA). R Studio version 1.1.463 (R Studio, Inc.) is utilized for data visualization. All the estimates provided in this study are derived by applying appropriate sampling weights supplied by NFHS-4, 2015–16.

## Results

### Description of the study population

Table 2 provides the descriptive and bivariate analysis findings for the sample of women (15–49 years) under consideration. The results suggest that 17.6% of the women were 15–19 years of age, 16.3% were 20–24 years of age, 15.8% were 25–29 years of age, 14.3% were 30–34 years of age, 13.5% were 35–39 years of age, 11.6% were 40–44 years of age and 11.1% were 45–49 years of age. Around 65.2% of the women belonged to rural areas, and 80.4% belonged to the Hindu religion. Around 43% of the women belonged to the OBC social group. Regarding education, 27% of the women surveyed were not educated, 13% completed primary education, 47% completed secondary education, and 13% completed higher schooling. It was found that 42% of the women belonged to the rich wealth quintile. Findings further depict that 71.9% of the women were living with their spouse during the survey period. The majority (56.8%) of the women in the age group 15–49 years had a parity of two or more. Around 4% of the respondents had experienced menopause. The majority of the women did not consume tobacco (93.08%) and alcohol (98.75%). Around 6% of the women were obese, whereas around 51% pursued an unhealthy dietary pattern.

**Table 2.** Sample distribution, prevalence of any one morbidity and multimorbidity, National Family Health Survey, India, 2015–16.

Correlates	Sample distribution (weighted percentage)	Prevalence rate (per 100 women)		Mean number of morbidities
		Any one morbidity	Two or more morbidities	
**Age (in years)**				
15–19	120285 (17.58)	7.66 (7.62-7.71)	0.54 (0.53-0.55)	0.09
20–24	108172 (16.32)	9.97 (9.91-10.03)	0.79 (0.78-0.81)	0.12
25–29	104472 (15.84)	13.50 (13.43-13.56)	1.58 (1.56-1.61)	0.17
30–34	92722 (14.30)	18.41 (18.33-18.49)	2.83 (2.80-2.87)	0.24
35–39	88969 (13.53)	22.81 (22.72-22.90)	4.77 (4.72-4.82)	0.33
40–44	75416 (11.60)	27.38 (27.28-27.48)	7.27 (7.21-7.34)	0.42
45–49	71775 (11.11)	31.18 (31.08-31.29)	10.26 (10.19-10.33)	0.52
		χ2 p-value=0.000		
**Place of** **residence**				
Urban	194801 (34.78)	18.64 (18.59-18.69)	4.46 (4.44-4.49)	0.28
Rural	467010 (65.22)	16.77 (16.73-16.81)	3.00 (2.97-3.01)	0.23
		χ2 p-value=0.000		
**Religion**				
Hindu	489798 (80.37)	17.03 (17.00-17.07)	3.31 (3.29-3.32)	0.23
Muslim	90183 (13.90)	18.70 (18.62-18.78)	4.27 (4.23-4.31)	0.28
Others	81830 (5.73)	19.72 (19.60-19.85)	4.31 (4.24-4.37)	0.29
		χ2 p-value=0.000		
**Social group**				
Scheduled Castes/ Tribes	239414 (29.72)	16.91 (16.86-16.97)	2.98 (2.96-3.00)	0.24
Other Backward Castes	255958 (42.98)	16.98 (16.94-17.03)	3.39 (3.37-3.41)	0.23
Others	166439 (27.30)	18.67 (18.61-18.72)	4.24 (4.21-4.27)	0.28
		χ2 p-value=0.000		
**Level of** **education**				
No education	180542 (26.62)	20.70 (20.64-20.76)	4.38 (4.31-4.37)	0.30
Primary	89028 (13.36)	19.78 (19.69-19.85)	4.37 (4.33-4.42)	0.28
Secondary	317411 (47.40)	15.67 (15.63-15.71)	3.04 (3.02-3.05)	0.22
Higher	74830 (12.62)	14.59 (14.52-14.68)	2.56 (2.52-2.59)	0.20
		χ2 p-value=0.300		
**Wealth index**				
Poorest	124542 (17.49)	16.06 (15.99-16.12)	2.32 (2.29-2.34)	0.21
Poorer	140851 (19.51)	16.16 (16.01-16.23)	2.76 (2.73-2.78)	0.22
Middle	139539 (20.57)	16.87 (16.80-16.93)	3.15 (3.12-3.18)	0.24
Richer	131845 (21.27)	18.63 (18.56-18.69)	4.27 (4.23-4.30)	0.27
Richest	125034 (21.15)	19.04 (18.97-19.11)	4.75 (4.71-4.78)	0.29
		χ2 p-value=0.000		
**Family status**				
Living without spouse	197488 (28.03)	11.29 (11.25-11.34)	1.67 (1.65-1.68)	0.15
Living with spouse	464323 (71.97)	19.81 (19.7-19.84)	4.22 (4.20-4.23)	0.29
		χ2 p-value=0.000		
**Parity**				
0	207921 (29.85)	10.14 (10.10-10.18)	1.21 (1.19-1.22)	0.13
1	82956 (13.35)	16.52 (16.45-16.60)	3.20 (3.16-3.24)	0.22
2 or more	370934 (56.81)	21.46 (21.42-21.50)	4.77 (4.75-4.80)	0.31
		χ2 p-value=0.000		
**Experienced** **menopause**				
No	634948 (95.98)	16.85 (16.82-16.88)	3.22 (3.21-3.23)	0.23
Yes	26863 (4.02)	31.07 (30.89-31.24)	10.29 (10.18-10.41)	0.52
		χ2 p-value=0.000		
**Tobacco** **consumption**				
No	591843 (93.08)	17.03 (17.00-17.06)	3.38 (3.37-3.39)	0.24
Yes	69968 (6.92)	22.69 (22.57-22.81)	5.17 (5.11-5.23)	0.33
		χ2 p-value=0.000		
**Alcohol** **consumption**				
No	645212 (98.75)	17.32 (17.29-17.35)	3.48 (3.46-3.49)	0.24
Yes	16599 (1.25)	25.21 (24.91-25.50)	5.53 (5.39-5.69)	0.34
		χ2 p-value=0.000		
**Body mass index** **(kg/m2)**				
Underweight	152490 (23.88)	11.79 (11.74-11.84)	1.31 (1.29-1.32)	0.15
Normal	370558 (53.08)	15.27 (15.23-15.31)	2.41 (2.39-2.42)	0.20
Overweight	105539 (17.13)	26.22 (26.14-26.31)	6.67 (6.61-6.70)	0.41
Obese	32930 (5.90)	33.99 (33.84-34.14)	13.08 (12.97-13.18)	0.64
		χ2 p-value=0.000		
**Diet type**				
Healthy	331072 (49.14)	16.46 (16.42-16.50)	3.16 (3.14-3.18)	0.23
Unhealthy	330739 (50.86)	18.35 (18.31-18.39)	3.83 (3.82-3.86)	0.26
		χ2 p-value=0.000		
**Total**	**6,61,811**	**17.42 (17.39-17.45)**	**3.49 (3.48-3.52)**	**0.25**

Findings from Table 2 further suggest that around 17.4 per 100 women in the reproductive age group suffered from any one morbidity, whereas around 3.5 per 100 women suffered from multimorbidity. All the variables, excluding education, were significantly associated with the burden of morbidity among women in the age group 15–49 years. The bivariate analysis suggests that the burden of any one morbidity and multimorbidity followed a similar pattern for all the selected background characteristics. The burden of morbidity (calculated by prevalence rate, PR) was found to follow an increasing trend with the age group of the respondent. It was found to be highest for women in the age group 45–49 years (PR: any one morbidity = 31.2 per 100 women; multimorbidity = 10.3 per 100 women). The burden was found to be higher for women residing in urban areas (PR: any one morbidity = 18.6 per 100 women; multimorbidity = 4.5 per 100 women), belonging to a religion other than Hindu and Muslim (PR: any one morbidity = 19.7 per 100 women; multimorbidity = 4.3 per 100 women), and from a social group other than Scheduled Castes/Tribes and OBC (PR: any one morbidity = 18.7 per 100 women; multimorbidity = 4.2 per 100 women). The burden was higher for a respondent who belonged to the well-off economic group (PR: any one morbidity = 18.7 per 100 women; multimorbidity = 4.2 per 100 women), was currently living with a spouse (PR: any one morbidity = 19.8 per 100 women; multimorbidity = 4.2 per 100 women), had a parity of two or more (PR: any one morbidity = 21.5 per 100 women; multimorbidity = 4.8 per 100 women), and had experienced menopause (PR: any one morbidity = 31.1 per 100 women; multimorbidity = 10.3 per 100 women). The burden of morbidity was found to be higher for women who consumed tobacco (PR: any one morbidity = 22.7 per 100 women; multimorbidity = 5.2 per 100 women), alcohol (PR: any one morbidity = 25.2 per 100 women; multimorbidity = 5.5 per 100 women), were obese (PR: any one morbidity = 33.4 per 100 women; multimorbidity = 13.1 per 100 women) and consumed an unhealthy diet (PR: any one morbidity = 18.3 per 100 women; multimorbidity = 3.8 per 100 women).

### Sub-national level variation in the burden of any one morbidity and multimorbidity

**Figure 1.**
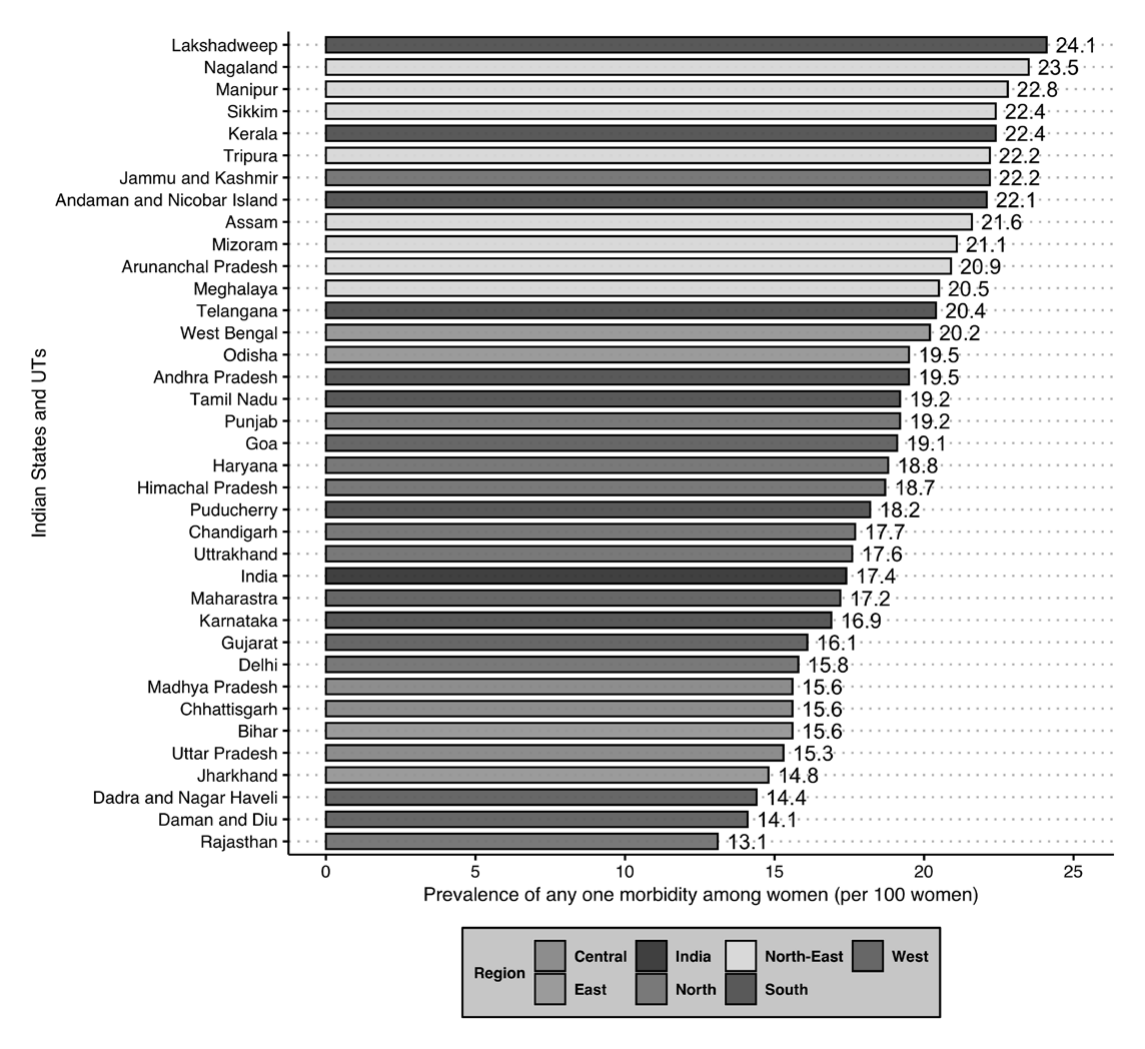
Prevalence of any one morbidity among women aged 15–49 years by States/Union Territories (UTs) in India, National Family Health Survey, 2015–16.

Figure 1 illustrates the burden of any one morbidity by all 36 states and UTs in India. The figure depicts that 24 states and UTs had a prevalence higher than that of the national average. Out of these 24 states, eight were from the North-Eastern, and seven were from the Southern parts of the country.

**Figure 2.**
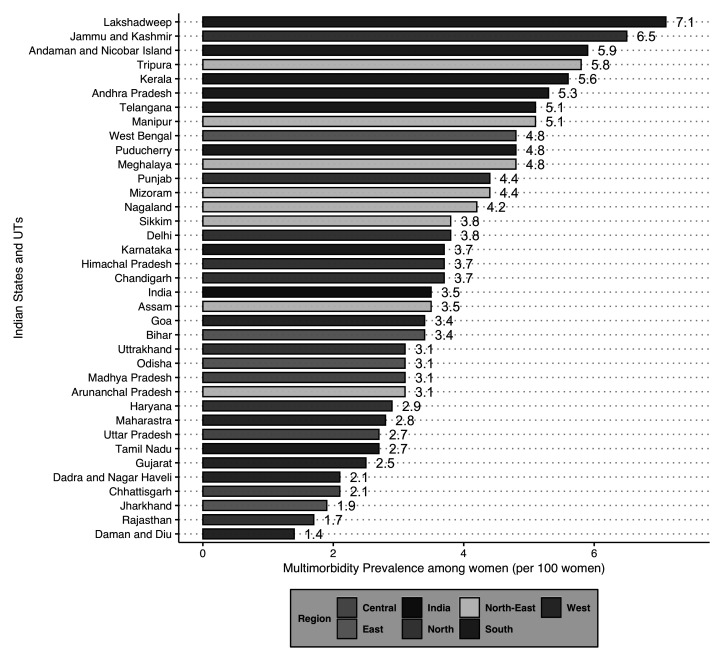
Prevalence of multimorbidity among women aged 15–49 years by States/Union Territories (UTs) in India, National Family Health Survey, 2015–16.

Figure 2 depicts the distribution of multimorbidity burden by all 36 states and UTs in India. The findings suggest that 19 states and UTs had a prevalence higher than that of the national average. Out of these 19 states and UTs, seven belonged to the Southern, and six belonged to the North-Eastern parts of the country.

### Variation in the burden of any one morbidity and multimorbidity by age group

**Figure 3.**
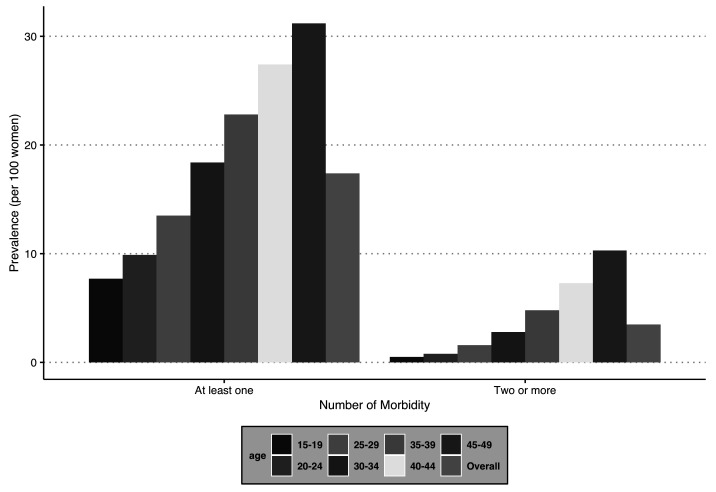
Prevalence of number of morbidities among women in India, National Family Health Survey, 2015–16.

Figure 3 provides the prevalence of any one morbidity and multimorbidity segregated by the selected age groups (in years). There is a trend observed in the burden (PR) of multimorbidity, which increases with the age of the women surveyed. A similar pattern is observed for any one morbidity and multimorbidity. The prevalence was lowest among the respondents in the age group 15–19 years (PR: any one morbidity = 7.7 per 100 women; multimorbidity = 0.5 per 100 women) and highest for the age group 45–49 years (PR: any one morbidity = 31.2 per 100 women; multimorbidity = 10.3 per 100 women).

### Pattern of the selected morbidities by age group

**Figure 4.**
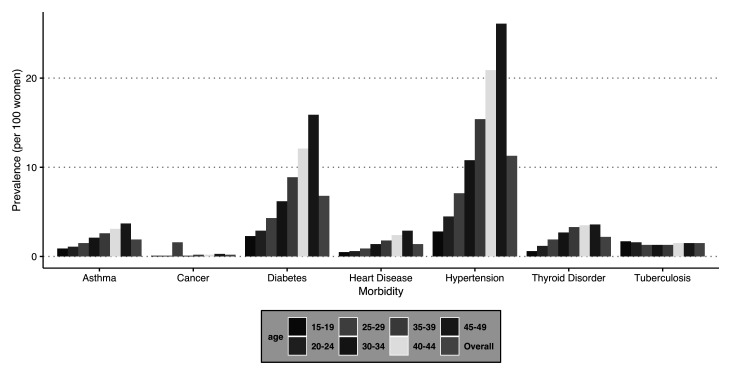
Prevalence of selected morbidities by age groups among women in India, National Family Health Survey, 2015–16.

The present study includes seven chronic conditions, namely, asthma, cancer, heart disease, diabetes, tuberculosis, hypertension, and thyroid disorder. Figure 4 depicts the prevalence of these selected chronic conditions segregated by age groups. All the morbidities show an increase with the age of the respondent. The findings further suggest that the most prevalent morbidity among women in the reproductive age group is hypertension (PR = 11.3%), followed by diabetes (PR = 6.8%) and thyroid disorder (PR = 2.2%) in India.

### Correlates of multimorbidity among women aged 15–49 years in India

Table 3 shows the adjusted effects of independent factors on the probability of suffering from multiple chronic morbidity conditions, i.e., multimorbidity using a two-part model. The predicted probability of having at least two chronic morbidity conditions reveals that the occurrence of multimorbidity is affected by different background characteristics.

**Table 3.** Two-part model estimates showing the factors affecting multimorbidity in India, National Family Health Survey, 2015–16.

Correlates	Respondent suffering with multimorbidity (predictive probabilities from logit model)	Average number of morbidities (predictive means from Poisson model)
**Age (in years)**		
15–19®	0.0055 ^***^±0.0004	0.0951 ^***^±0.0012
20–24	0.0078 ^***^±0.0004	0.1226 ^***^±0.0012
25–29	0.0146 ^***^±0.0006	0.1671 ^*** ^±0.0013
30–34	0.0244 ^***^±0.0008	0.2294 ^***^±0.0017
35–39	0.0392 ^***^±0.0010	0.3014 ^***^±0.0020
40–44	0.0575 ^***^±0.0011	0.3747 ^***^±0.0003
45–49	0.0781 ^***^±0.0020	0.4445 ^***^±0.0003
**Place of residence**		
Urban®	0.0201±0.0005	0.1969±0.0010
Rural	0.0192±0.0003	0.1982±0.0007
**Religion**		
Hindu®	0.0188±0.0003	0.1905±0.0007
Muslim	0.0235 ^***^±0.0007	0.2277 ^***^±0.0016
Others	0.0206 ^*^±0.0005	0.2061 ^***^±0.0011
**Social group**		
Scheduled Castes/ Tribes®	0.0194±0.0005	0.1999±0.0009
Other Backward Castes	0.0188±0.0004	0.1907 ^***^±0.0008
Others	0.0206±0.0005	0.2061 ^***^±0.0011
**Level of education**		
No education®	0.0192±0.0004	0.1950±0.0011
Primary	0.0188±0.0004	0.2075±0.0014
Secondary	0.0200±0.0004	0.2014±0.0009
Higher	0.0163±0.0007	0.1792±0.0007
**Wealth index**		
Poorest®	0.0195±0.0006	0.1929±0.0014
Poorer	0.0215±0.0006	0.1972±0.0012
Middle	0.0200±0.0004	0.1956±0.0014
Richer	0.0212 ^***^±0.0005	0.2027 ^***^±0.0012
Richest	0.0206 ^***^±0.0006	0.2009 ^***^±0.0014
**Family status**		
Living without spouse	0.0189±0.00060	0.1966±0.0006
Living with spouse®	0.0197±0.0004	0.2288±0.0021
**Parity**		
0®	0.0234±0.0008	0.2136±0.0017
1	0.0245 ^***^±0.0006	0.2281 ^***^±0.0016
2 or more	0.0336 ^***^±0.0004	0.2494 ^***^±0.0009
**Experienced** **menopause**		
No	0.0193±0.0003	0.1966±0.0006
Yes	0.0228 ^*^±0.0008	0.2288 ^***^±0.0021
**Tobacco** **consumption**		
No®	0.0192±0.0003	0.1957±0.0006
Yes	0.0251 ^***^±0.0009	0.2288 ^***^±0.0021
**Alcohol** **consumption**		
No®	0.0194±0.0003	0.1970±0.0006
Yes	0.02282 ^***^±0.0008	0.2288 ^***^±0.0031
**Body mass index** **(kg/m2)**		
Underweight®	0.01227±0.0004	0.1588±0.0011
Normal	0.0174 ^***^±0.0003	0.1823 ^***^±0.0007
Overweight	0.0344 ^***^±0.0008	0.2868 ^***^±0.0016
Obese	0.6345 ^***^±0.0019	0.4158 ^***^±0.0031
**Diet type**		
Healthy®	0.0179±0.0003	0.1894±0.0008
Unhealthy	0.0211 ^***^±0.0004	0.2066 ^***^±0.0008
**Total**	0.01945 ^***^±0.0003	0.1978 ^***^±0.006

Note. *p<0.05, **p<0.01, ***p<0.001, ± value of predicted probabilities or means calculated as upper limit-lower limit of 95% confidence interval divided by 2. ® = reference.

For women of reproductive age, the predictive probability shows that variables such as age (in years), religion, wealth index, parity, experienced menopause, consumption of tobacco, consumption of alcohol, BMI, and type of diet are statistically significant predictors of multimorbidity.

The findings suggest that a shift in the age group from 15–19 years to 20–24 years, 25–29 years, 30–34 years, 40–44 years and, 45–49 years increases the probability of suffering from multimorbidity by 0.2%, 0.9%, 1.9%, 3.4%, 5.2%, and 7.3% respectively, after controlling for other background characteristics.

The findings suggest that as an individual moves from Hindu to Muslim, the probability of suffering from multimorbidity increases by 0.5% after controlling for other background factors. An increase in wealth index from the poorest to richest increases the probability of suffering from multimorbidity by 0.1%.

The probability of suffering from multimorbidity increases by 0.1% as parity increases from zero to one. The probability of suffering from multimorbidity increases by 0.9% as parity increases from zero to two or more. Similarly, the probability of suffering from multimorbidity among women who have experienced menopause is higher by 0.4% as compared to those who have not experienced menopause.

A shift of a woman to consuming tobacco and alcohol from not consuming tobacco and alcohol increases the probability of suffering from multimorbidity by 0.6% and 0.3%, respectively. The findings suggest that a shift in women from underweight to obese increases the probability of suffering from multimorbidity by 62% after controlling for key factors. A shift of women from following a healthy diet to an unhealthy diet increases the probability of suffering from multimorbidity by 0.3%.

## Discussion

Based on the data from the fourth round of NFHS, 17.4 per 100 women in the reproductive age group suffered from any one morbidity, whereas 3.5 per 100 women suffered from multimorbidity (two or more morbidities). Findings further suggest that a regional disparity exists in the multimorbidity burden among women in the reproductive age group, with the Southern and North-Eastern regions of the country experiencing a higher burden of multimorbidity. Hypertension, diabetes, and thyroid disorders were commonly occurring morbidities among women of reproductive age. The prevalence of having any one morbidity and multimorbidity increased with age. At the later stages of their reproductive span (45–49 years), the women held the highest burden of morbidity. Variables like religion, wealth, parity, menopause, consumption of tobacco and alcohol, BMI, and type of diet were significantly related to the burden of multimorbidity among women of child-bearing age. 

A recent systematic review conducted on the studies based on low-and-middle-income countries (LMICs) suggests that the prevalence of multimorbidity ranges between 2% to 82% for LMICs ( Marengoni *et al.*, 2011). Findings from the present study show that the prevalence of any one morbidity and multimorbidity among women in India’s reproductive age group is 17.4% and 3.5%, respectively, which falls in the range of the above research. The study’s findings depict a large proportion of women in their reproductive years suffering from chronic morbidities. Presently running women’s health programs in India are mainly focused on sexual and reproductive health care ( National Health Portal, 2005; Government of India, 2016; Government of India, 2017). However, the increasing level of morbidity burden among women of reproductive age needs to be given attention from a policy point of view. 

The burden of multimorbidity was found to be higher for respondents belonging to the Southern and the North-Eastern regions of India. This could be attributed to two reasons, one being the differing lifestyles and second, the nutritional transition in the country ( NCD Risk Factor Collaboration (NCD-RisC), 2017; Jovic *et al.*, 2016). Studies in the past suggest that dietary pattern in India is extremely diverse, which encompasses a prudent/traditional and western pattern ( Green *et al.,* 2016; Satija *et al.*, 2015). However, these two sub-groups are not mutually exclusive to each other. The prudent pattern comprises of a diet which is high in fruits, vegetables, legumes, fish, dairy products, and wholegrains ( Green *et al.*, 2016; Satija *et al.*, 2015). In contrast, a western pattern is high in processed meat, eggs, refined grains, sugar, and fast food ( Green *et al.*, 2016). Existing evidence suggests that the western pattern diet affects the body size of an individual, which further increases the risk of chronic conditions like diabetes and hypertension ( Kehoe *et al.*, 2014; Satija *et al.*, 2015). Studies discussing the dietary pattern by different regions of India suggest that the Southern region of India mainly follows two types of diets, namely “rice-based” ( Joy *et al.*, 2017; Satija *et al.*, 2015), which is most prevalent among adults, and “snack-fruit-based” ( Kehoe *et al.*, 2014), which is most frequent among children. This diet includes snacks that are usually high in fats and salt. These studies further point out that long term exposure to these diets results in increased adiposity, which further accelerates the risk of being affected by chronic morbidities ( Green *et al.*, 2016; Satija *et al.*, 2015). The results presented by studies discussing the higher morbidity rates in North-East India were mainly linked to the behavioural aspects of the region, which includes higher consumption of tobacco, alcohol, and other materials of substance abuse ( Bhar *et al.,* 2019; Tushi *et al.,* 2018).

Evidence generated by this study suggests a preponderance of multimorbidity among women in the later stage of their reproductive years. It is worth mentioning that hypertension, diabetes, and thyroid are common morbidities among the women surveyed. This could be due to the depletion of estrogen levels that occurs as women reach the later years of their reproductive stage, which is strongly linked to their reproductive capabilities. Existing literature suggests that this reduction in estrogen levels causes alterations in the biological well-being of women and increases the risk of morbidity occurrence. The findings can further be generalised for all women of reproductive age in India. The factors of parity and whether menopause had been experienced also suggest that the chance of being affected by multimorbidity increases as the reproductive exposure increases. These findings are in line with studies done in different country settings ( Bhar *et al.*, 2019; Levine *et al.*, 2016; Xu *et al.*, 2020).

The prevalence of multimorbidity was found to be higher for respondents belonging to economically well-off groups of society. This finding is similar to that of other studies that have been conducted in LMICs. The main reason behind this finding is the fact that with economic liberalisation, globalisation, and westernisation, the dietary pattern of the population is changing, the consumption of food and beverages rich in saturated sugar is increasing, and the number of individuals practicing a sedentary lifestyle are also increasing considerably ( NCD Risk Factor Collaboration (NCD-RisC), 2017; Green *et al.*, 2016).

 Additionally, consuming tobacco, consuming alcohol, being obese, and consuming an unhealthy diet were found to be associated with a higher multimorbidity burden among women. This finding is in concordance with the existing literature, which establishes the consumption of tobacco and alcohol as the major correlates of various chronic conditions ( Amos *et al.,* 2012; Lin *et al.,* 2007; Singh & Singh, 2016). Existing literature has presented a negative association between an unhealthy diet and the occurrence of chronic diseases ( Satija *et al.*, 2015; Shridhar *et al.*, 2015). These studies suggest that a diet rich in salt, sugar, and trans-fats often accelerates metabolic risk and adiposity, which further results in the occurrence of one or more chronic conditions ( Kehoe *et al.*, 2014; Shridhar *et al.*, 2015). A recent study proposes maintaining dietary adherence to control diabetes among individuals residing in the United States of America, similar recommendations can be given to manage multimorbidity among women of reproductive age in India ( Anders & Schroeter, 2015).

The study aims to explore the pattern and correlates of multimorbidity among women of reproductive age in India. The study’s major strength is that it is based on large-scale nationally representative cross-sectional data, which provides us with the opportunity to generalize the results for all the women of reproductive age in India. The studies done so far in the context of women of reproductive age were mainly focused on sexual and reproductive health aspects. However, the rising levels of various infectious and non-communicable morbidities among women of child-bearing age have not been studied in the Indian context, necessitating the present study. The study findings suggest linkages between reproductive exposures and the occurrence of multimorbidity among women of reproductive age, which, so far, have not been discussed in the context of India. However, the present study does not include a large number of chronic conditions (only seven conditions were included) or variations of the chronic diseases, thus missing out on the vital aspect of mental health as NFHS does not provide this information.

The study’s findings help us understand that SES, reproductive exposures, and behavioral factors play a vital role in the occurrence of one or more chronic morbidities among women in their reproductive age group. Considering the points above, it becomes essential to provide personalized age-specific healthcare facilities to the women affected by one or multiple morbidities ( Fortin *et al.*, 2004; Monterde *et al.*, 2020). In addition, educating women regarding the importance of maintaining a healthy lifestyle, an ideal body weight, and dietary diversity is crucial, as reproductive years are the foundation of their health in later life. However, this would require an in-depth study considering more chronic conditions specific to women from all age groups in India.

The present study suggests that one or multiple morbidities are important in women of reproductive age. The findings necessitate further exploration of the issue, especially linkages between various chronic conditions in a life course perspective. Inclusion of chronic disease management strategies with maternal and child health services needs to be considered by the program and policymakers. Additionally, social marketing approaches at the primary level of healthcare would help policymakers educate women about the importance of leading a healthy lifestyle. Practicing dietary diversity can help maintain optimal estrogen levels, which would further help to decrease multimorbidity rates among women in India.

## Data availability

The data has been archived in the public repository of the Demographic and Health Survey of India. The present study utilized information from individual recode file IAIR74DT, which essentially contains information on women in the reproductive age group. The data can be accessed using the link: https://www.dhsprogram.com/data/dataset/India_Standard-DHS_2015.cfm?flag=0. Access to the dataset requires registration and is granted only for legitimate research purposes. A guide for how to apply for dataset access is available at: https://dhsprogram.com/data/Access-Instructions.cfm.
