# Supplementary material for: Burden and determinants of multimorbidity among women in reproductive age group: a cross-sectional study based in India
Source: Wellcome Open Res. 2021 Feb 18;5:275. Originally published 2020 Nov 24. [Version 2] doi: 10.12688/wellcomeopenres.16398.2 (PMC8182697; doi:10.12688/wellcomeopenres.16398.2)
Supplement: Peer review history

# Peer review: Reviewer response for version 2

**DOI:** 10.21956/wellcomeopenres.18185.r44069

**Published:** 2021-06-04

**Authors:** Srinivas Goli

**Affiliations:** 1 The University of Western Australia, Crawley, WA, Australia; 2 New Generation Network Scholar, Melbourne, Australia

The present study explores the burden and predictors of multimorbidity among women in the reproductive age group (15-49 years) utilizing a nationally representative data from the recent round of National Family Health Survey (NFHS), 2015-16, India. This is an interesting work, however, there are a few observations, which can improve the overall quality of the research work:

1. **Page 4, Variables description:** The authors mention measuring internal consistency using Cronbach’s alpha, however, the value of Cronbach’s alpha is not mentioned anywhere in the text. Was the value in the acceptable range?
2. **Page 4, Variables description:** The authors mention checking for multicollinearity twice in the variables section. It would be appropriate to keep it once at the end of the variable section.
3. **Page 5, Statistical Analysis: **The authors used a two-part model in the study, however, the justification provided to use the same is quite lengthy and repetitive. Readers would benefit from tightening it.
4. **Page 6, Methods Section: **There are some repetitions in the description of software use. Kindly check and correct that.
5. Other Backward Class (OBC) is a collective term used by the Government of India to classify castes which are educationally or socially disadvantaged. The authors have however used Other Backward Caste in the tables, which needs to be rectified.
6. **Page 8, Result Section:** Considering the geographical variations, despite of giving estimates for both states and Union Territories (UTs), is it appropriate to give the description of only state and not UTs?
7. **Page 11, Result Section:** Considering the Body Mass Index (BMI), it would make more sense if normal BMI should be taken as a reference category rather than underweight. Please modify the same in the revised manuscript.
8. Check the entire results section for repetitions and a vigorous proof-reading of the manuscript will be useful.

Is the work clearly and accurately presented and does it cite the current literature?

Yes

If applicable, is the statistical analysis and its interpretation appropriate?

Yes

Are all the source data underlying the results available to ensure full reproducibility?

Yes

Is the study design appropriate and is the work technically sound?

Yes

Are the conclusions drawn adequately supported by the results?

Yes

Are sufficient details of methods and analysis provided to allow replication by others?

Yes

Reviewer Expertise:

Public Health, Nutrition and Ageing

I confirm that I have read this submission and believe that I have an appropriate level of expertise to confirm that it is of an acceptable scientific standard.

# Peer review: Reviewer response for version 2

**DOI:** 10.21956/wellcomeopenres.18185.r42678

**Published:** 2021-02-22

**Authors:** Ram Bajpai

**Affiliations:** 1 School of Medicine, Keele University, Keele, UK

Authors have appropriately responded on my previous comments. I have no further questions.

Is the work clearly and accurately presented and does it cite the current literature?

Yes

If applicable, is the statistical analysis and its interpretation appropriate?

Yes

Are all the source data underlying the results available to ensure full reproducibility?

Yes

Is the study design appropriate and is the work technically sound?

Yes

Are the conclusions drawn adequately supported by the results?

Yes

Are sufficient details of methods and analysis provided to allow replication by others?

Yes

Reviewer Expertise:

Epidemiology, Biostatistics, Public health

I confirm that I have read this submission and believe that I have an appropriate level of expertise to confirm that it is of an acceptable scientific standard.

# Peer review: Reviewer response for version 1

**DOI:** 10.21956/wellcomeopenres.18038.r41592

**Published:** 2020-12-03

**Authors:** Ram Bajpai

**Affiliations:** 1 School of Medicine, Keele University, Keele, UK

Authors have well designed and presented their research work. However, the following suggestion will help further to improve the quality of this report.

 Why did authors use international criteria-based BMI rather than Asian criteria-based BMI (<18.5 for underweight, 18.5-22.9 for normal-weight, 23.0-27.5 for overweight, and >27.5 for obese) for women in this report? Some justification will be very helpful to wider audience of this report.

 Negative binomial regression and hurdle models can also be used for similar purpose to model over-dispersed count outcome variables. How authors did preferred two-part model over the hurdle models and negative binomial regression? A proper explanation is solicited from authors with citations in the analysis plan section.

 The fit for each of two equations can be assessed with conventional tests and approaches as suggested in the literature. So, how goodness-of-fit was checked for two-part-model (such as deviance distribution, correlation between observed and predicted outcome, and link test for model adequacy)?

 How were colinearity and interactions between predictors and outcomes assessed and controlled in the model? It should have been explained in the analysis plan.

 The p-value statement is missing in the statistical analysis plan. Please add that.

**Table 2** - reporting just mean number of morbidities is not helpful as the data were highly skewed. Authors should consider median with IQR for those reported morbidity and they can highlight it in the table footnote that it was calculated for non-zero morbidity group. Authors may also find other appropriate way to present this data. Please do not report ‘p-value=0.000’, a better approach would be to report it as ‘p<0.001’.

**Figures 1 and 2** - is it possible to present India in bold at the bottom? Can you also put them side-by-side in one figure for better comparison?

**Table 3** - presents predictive probabilities/means along with respective standard errors (please mention it). Is it possible to estimate and present ORs (with 95% CI) for first part and PRs (with 95% CI) to the second part, as it will be easy to interpret results from the regression model?

 No study is free of limitations. So, what are the study limitations, as I could not find any in this report? Kindly report as appropriate.

Is the work clearly and accurately presented and does it cite the current literature?

Yes

If applicable, is the statistical analysis and its interpretation appropriate?

Yes

Are all the source data underlying the results available to ensure full reproducibility?

Yes

Is the study design appropriate and is the work technically sound?

Yes

Are the conclusions drawn adequately supported by the results?

Yes

Are sufficient details of methods and analysis provided to allow replication by others?

Yes

Reviewer Expertise:

Epidemiology, Biostatistics, Public health

I confirm that I have read this submission and believe that I have an appropriate level of expertise to confirm that it is of an acceptable scientific standard, however I have significant reservations, as outlined above.

# Response

**Published:** 2020-12-09

**Authors:** Sanghamitra Pati

**Affiliations:** ICMR RMRC Bhubaneswar, India

**Reviewer Comments, Author Responses and Manuscript Changes**

**Comment #1** **:** Why did authors use international criteria-based BMI rather than Asian criteria-based BMI (<18.5 for underweight, 18.5-22.9 for normal-weight, 23.0-27.5 for overweight, and >27.5 for obese) for women in this report? Some justification will be very helpful to wider audience of this report.

**Reply #1:** Thank you for the observation. The authors utilized information from the fourth round of the National Family Health Survey (NFHS-4), 2015-16, which falls under Demographic and Health Surveys (DHS). In the present article, the authors have utilized information on selected anthropometric and clinical diagnostic tests; thus, to maintain consistency which the DHS guidelines, the authors used the cut-off suggested by the "Guide to DHS Statistics-2018". The cut-offs mentioned in the DHS guide has been widely accepted. The guide can be accessed using: https://dhsprogram.com/pubs/pdf/DHSG1/Guide_to_DHS_Statistics_DHS-7.pdf.

**Comment #2:** Negative binomial regression and hurdle models can also be used for similar purpose to model over-dispersed count outcome variables. How authors did preferred two-part model over the hurdle models and negative binomial regression? A proper explanation is solicited from authors with citations in the analysis plan section.

**Reply #2: ** Thank you for the observation. We were interested in identifying the patterns of one or more morbidities and to examine the correlates of multimorbidity among women in the reproductive age group. While examining the correlates of multimorbidity, we have conceptualized the research question in such a way that, we first identify whether a woman is affected with multimorbidity or not, further we aimed to identify what are the average number of morbidities in any individual affected with multimorbidity. Also, whether or not there is any significant difference between the number of morbidities by background characteristics. For this purpose, we have utilized a two-part model over other models.  A complete description is provided with reference in the analysis, which can be seen on page 10, line number 211-221.

**Comment #3** **:** The fit for each of two equations can be assessed with conventional tests and approaches as suggested in the literature. So, how goodness-of-fit was checked for two-part-model (such as deviance distribution, correlation between observed and predicted outcome, and link test for model adequacy)?

**Reply #3** **: **A Hosmer-Lemeshow and chi-square goodness of fit test were used to establish model adequacy for logit (https://www.stata.com/manuals13/restatgof.pdf) and poission’ model (https://stats.idre.ucla.edu/stata/dae/poisson-regression/), respectively. The goodness of fir statistics are presented in Table 3. Also, this part has been added in the statistical analysis section on page 10, line 222-224.

**Comment #4** **:** How were collinearity and interactions between predictors and outcomes assessed and controlled in the model? It should have been explained in the analysis plan.

**Reply #4** **: **Initially a range of socioeconomic, reproductive and lifestyle predictors were included in the study. However, after checking the Variance Inflation Factor (VIF) few reproductive variables were dropped (VIF>=10.0) from the final model. This is now highlighted in the revised version of the manuscript and can be found on page 7, line 158-160.

**Comment #5** **:** The p-value statement is missing in the statistical analysis plan. Please add that.

**Reply #5:** The p-value statement has been added as suggested by the reviewer. The changes can be found on page 10, line 221.

**Comment #6** **:** Table 2 - reporting just mean number of morbidities is not helpful as the data were highly skewed. Authors should consider median with IQR for those reported morbidity and they can highlight it in the table footnote that it was calculated for non-zero morbidity group. Authors may also find other appropriate way to present this data. Please do not report ‘p-value=0.000’, a better approach would be to report it as ‘p<0.001’.

**Reply #6** **: **Thank you for the suggestion. We have made all the changes as suggested, which can be seen in the Table 2.

**Comment #7:** Figures 1 and 2 - is it possible to present India in bold at the bottom? Can you also put them side-by-side in one figure for better comparison?

**Reply #7** **: **Thank you for the suggestion, the figures are now kept side-by-side as suggested by the reviewer. Further, figures 1 and 2, states/union territories are placed in a descending order of prevalence of any-one and multimorbidity, so that their comparative position with respect to the national average (India) is clearly visible.

**Comment #8:** Table 3 - presents predictive probabilities/means along with respective standard errors (please mention it). Is it possible to estimate and present ORs (with 95% CI) for first part and PRs (with 95% CI) to the second part, as it will be easy to interpret results from the regression model?

**Reply #8:** This comment has been extremely insightful. In the Table 3 we have now replaced the existing predictive probabilities in the poisson’ regression with predictive means (average number of chronic conditions) in individual affected with multimorbidity. Both predictive probabilities (in logistic regression) and predictive means are substituted with Standard Error (S.E.) as suggested by the reviewer.  

**Comment #9:** No study is free of limitations. So, what are the study limitations, as I could not find any in this report? Kindly report as appropriate.

**Reply #9:** Thank you for the observation. The major limitation of the present study is that it does not include a large number of chronic conditions (only seven conditions were included) or variations of the chronic diseases, thus missing out on the vital aspect of mental health as NFHS does not provide this information. However, adhering to the journal- *Wellcome Open Research’s *author guidelines, we could not add a separate section on strengths and limitations of the study, therefore the aforementioned paragraph was added in the discussion section of the manuscript. The limitation can be found on page 18, line 415-418.
